# Supplementary material for: Comprehensive multiomic characterization of human papillomavirus-driven recurrent respiratory papillomatosis reveals distinct molecular subtypes
Source: Commun Biol. 2021 Dec 20;4:1416. doi: 10.1038/s42003-021-02942-0 (PMC8688513; doi:10.1038/s42003-021-02942-0)
Supplement: Supplementary file 2 — Supplementary Information [file 42003_2021_2942_MOESM2_ESM.pdf]

Supplementary Figures & Legends & Table

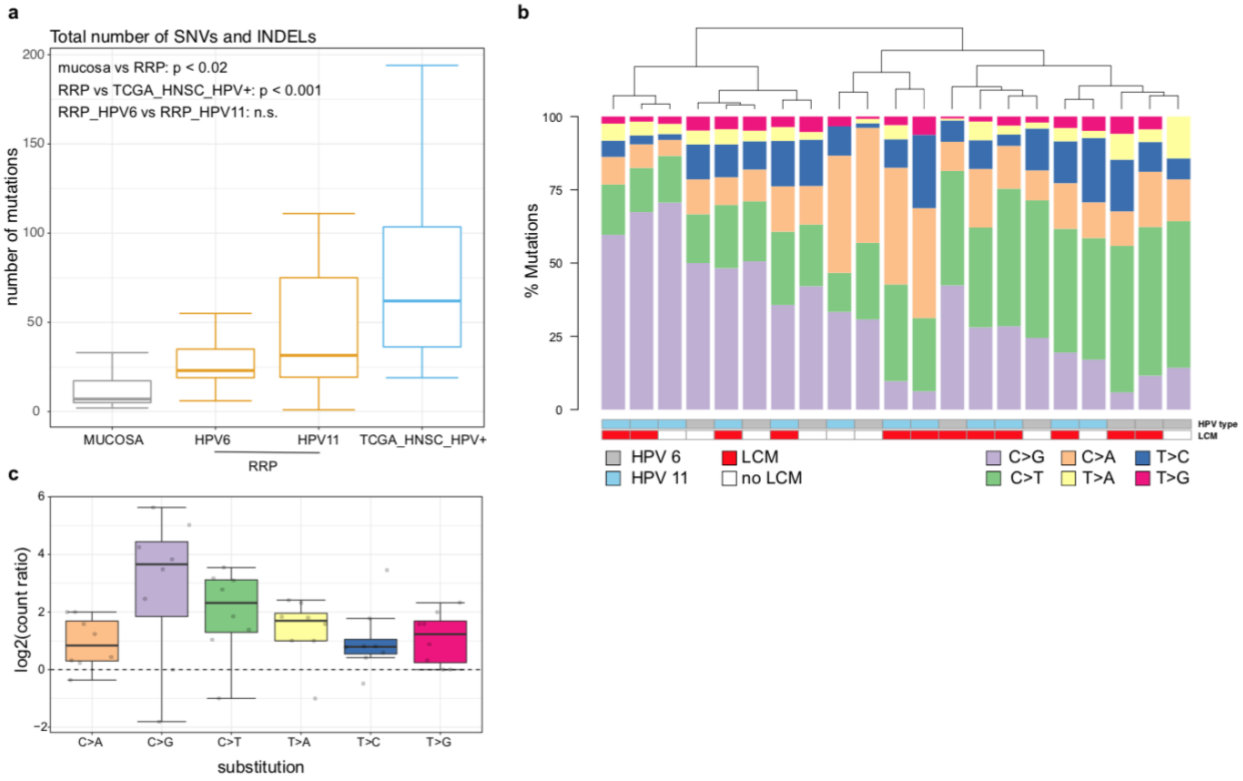

Supplementary Figure 1 - Somatic variants identified in RRP and normal mucosa

a, boxplot shows the total number of somatic variants, i.e. SNVs and INDELs, identified within normal mucosa (*n*=8), RRP (*n*=21) and HPV-associated HNSCC (*n*=74).

b, barplot shows the fraction of specific substitutions observed within individual RRP samples (columns). Hierarchical clustering revealed three main clusters that differed by their most frequent substitution (C to G, C to A and C to T). HPV type and LCM processing status are shown below each sample.

c, boxplot shows the log2 count fold change of specific substitutions in RRP relative to matched mucosa (*n*=8). Only RRP samples with matched normal mucosa are shown.

SNV, single nucleotide variation; INDEL, small insertion or deletion; LCM, laser-capture microdissection

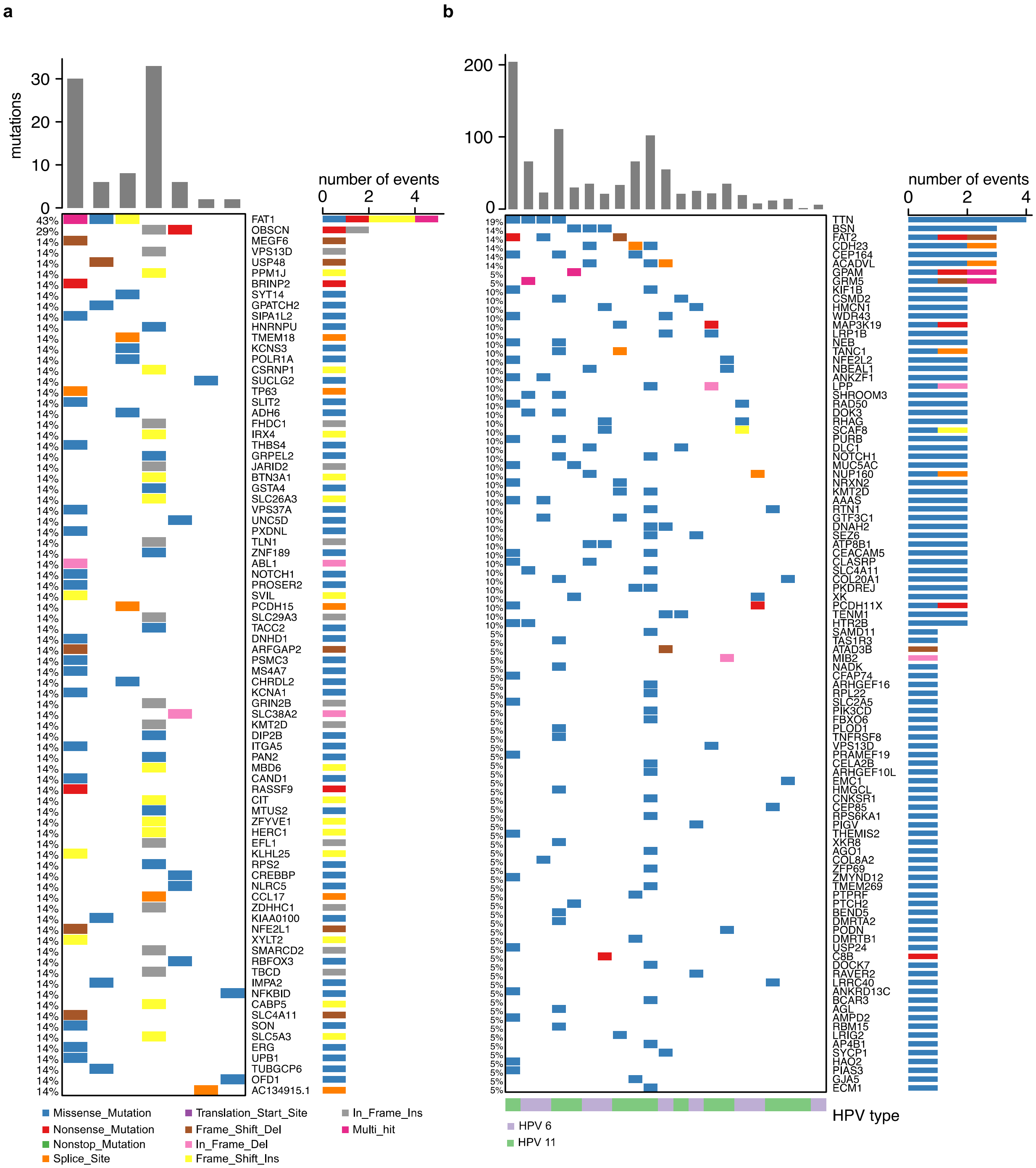
a
b
mutations
30
20
10
0
200
100
number of events
0
2
4
FAT1
OBSCN
MEGF6
VPS13D
USP48
PPM1J
BRINP2
SYT14
GPATCH2
SIPA1L2
HNRNPU
TMEM18
KCNS3
POLR1A
CSRNP1
SUCLG2
TP63
SLIT2
ADH6
FHDC1
IRX4
THBS4
GRPEL2
JARID2
BTN3A1
GSTA4
SLC26A3
VPS37A
UNC5D
PXDNL
TLN1
ZNF189
ABL1
NOTCH1
PROSER2
SVIL
PCDH15
SLC29A3
TACC2
DNHD1
ARFGAP2
PSMC3
MS4A7
CHRDL2
KCNA1
GRIN2B
SLC38A2
KMT2D
DIP2B
ITGA5
PAN2
MBD6
CAND1
RASSF9
CIT
MTUS2
ZFYVE1
HERC1
EFL1
KLHL25
RPS2
CREBBP
NLRC5
CCL17
ZDHHC1
KIAA0100
NFE2L1
XYLT2
SMARCD2
RBFOX3
TBCD
IMPA2
NFKBID
CABP5
SLC4A11
SON
SLC5A3
ERG
UPB1
TUBGCP6
OFD1
AC134915.1
TTN
BSN
FAT2
CDH23
CEP164
ACADVL
GPAM
GRM5
KIF1B
CSMD2
HMCN1
WDR43
MAP3K19
LRP1B
NEB
TANC1
NFE2L2
NBEAL1
ANKZF1
LPP
SHROOM3
RAD50
DOK3
RHAG
SCAF8
PURB
DLC1
NOTCH1
MUC5AC
NUP160
NRXN2
KMT2D
AAAS
RTN1
GTF3C1
DNAH2
SEZ6
ATP8B1
CEACAM5
CLASRP
SLC4A11
COL20A1
PKDREJ
XK
PCDH11X
TENM1
HTR2B
SAMD11
TAS1R3
ATAD3B
MIB2
NADK
CFAP74
ARHGEF16
RPL22
SLC2A5
PIK3CD
FBXO6
PLOD1
TNFRSF8
VPS13D
PRAMEF19
CELA2B
ARHGEF10L
EMC1
HMGCL
CNKSR1
CEP85
RPS6KA1
PIGV
THEMIS2
XKR8
AGO1
COL8A2
ZFP69
ZMYND12
TMEM269
PTPRF
PTCH2
BEND5
DMRTA2
PODN
DMRTB1
USP24
C8B
DOCK7
RAVER2
LRRC40
BCAR3
AGL
AMPD2
RBM15
LRIG2
AP4B1
SYCP1
HAO2
PIAS3
GJA5
ECM1
Missense_Mutation
Nonsense_Mutation
Nonstop_Mutation
Splice_Site
Translation_Start_Site
Frame_Shift_Del
In_Frame_Del
Frame_Shift_Ins
In_Frame_Ins
Multi_hit
HPV type
HPV 6
HPV 11

Supplementary Figure 2 - Quantification of mutation counts and base substitutions.

Heatmaps show mutations within genes with the highest mutation frequency (rows) in **a**, normal mucosa (*n*=8) and **b**, RRP samples (*n*=21). The barplots above each heatmap show the total number of mutations within each sample. HPV type is shown below in **b**.

a

b

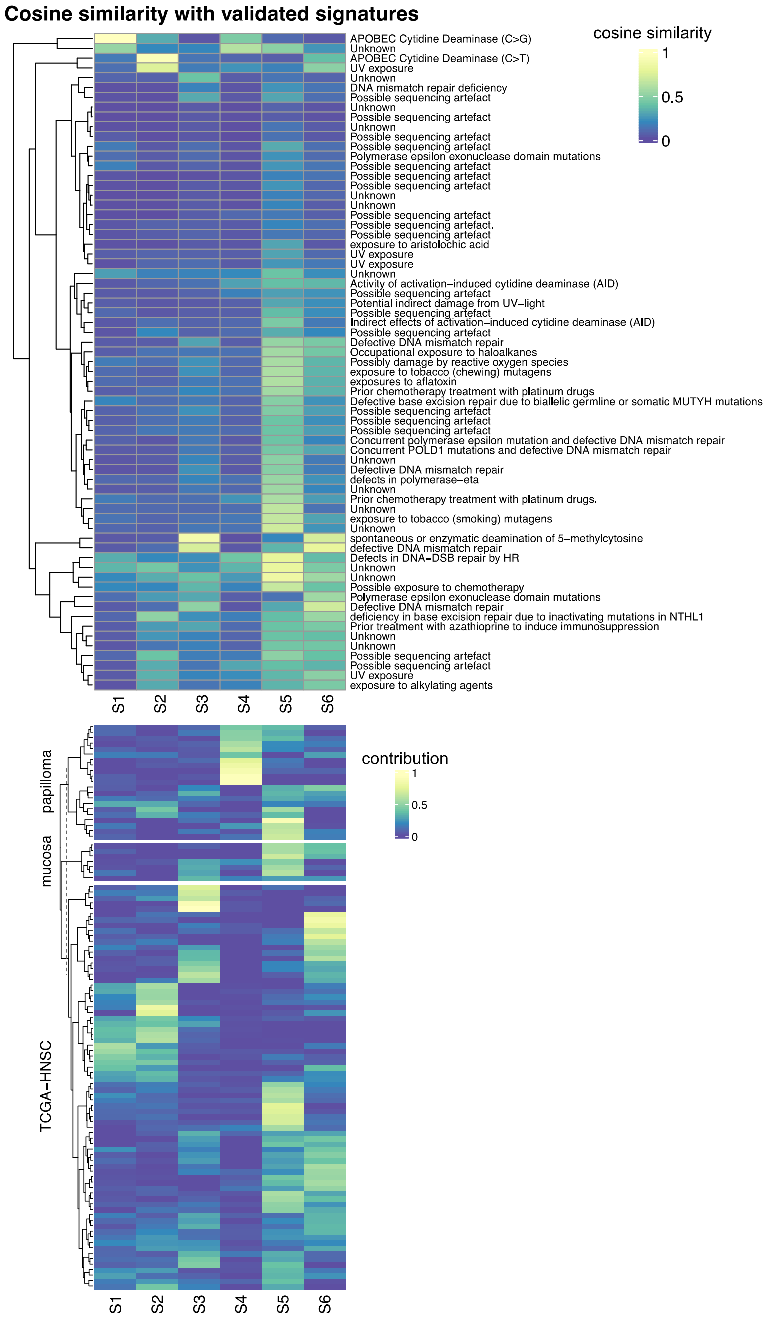

Cosine similarity with validated signatures
cosine similarity
1
0.5
0
APOBEC Cytidine Deaminase (C>G)
Unknown
APOBEC Cytidine Deaminase (C>T)
UV exposure
Unknown
DNA mismatch repair deficiency
Possible sequencing artefact
Unknown
Possible sequencing artefact
Unknown
Possible sequencing artefact
Possible sequencing artefact
Polymerase epsilon exonuclease domain mutations
Possible sequencing artefact
Possible sequencing artefact
Possible sequencing artefact
Unknown
Unknown
Possible sequencing artefact
Possible sequencing artefact.
Possible sequencing artefact
exposure to aristolochic acid
UV exposure
UV exposure
Unknown
Activity of activation−induced cytidine deaminase (AID)
Possible sequencing artefact
Potential indirect damage from UV−light
Possible sequencing artefact
Indirect effects of activation−induced cytidine deaminase (AID)
Possible sequencing artefact
Defective DNA mismatch repair
Occupational exposure to haloalkanes
Possibly damage by reactive oxygen species
exposure to tobacco (chewing) mutagens
exposures to aflatoxin
Prior chemotherapy treatment with platinum drugs
Defective base excision repair due to biallelic germline or somatic MUTYH mutations
Possible sequencing artefact
Possible sequencing artefact
Possible sequencing artefact
Concurrent polymerase epsilon mutation and defective DNA mismatch repair
Concurrent POLD1 mutations and defective DNA mismatch repair
Unknown
Defective DNA mismatch repair
defects in polymerase−eta
Unknown
Prior chemotherapy treatment with platinum drugs.
Unknown
exposure to tobacco (smoking) mutagens
Unknown
spontaneous or enzymatic deamination of 5−methylcytosine
defective DNA mismatch repair
Defects in DNA−DSB repair by HR
Unknown
Unknown
Possible exposure to chemotherapy
Polymerase epsilon exonuclease domain mutations
Defective DNA mismatch repair
deficiency in base excision repair due to inactivating mutations in NTHL1
Prior treatment with azathioprine to induce immunosuppression
Unknown
Unknown
Possible sequencing artefact
Possible sequencing artefact
UV exposure
exposure to alkylating agents
S1
S2
S3
S4
S5
S6
papilloma
mucosa
TCGA−HNSC
contribution
1
0.5
0
S1
S2
S3
S4
S5
S6

Supplementary Figure 3 - Non-negative matrix factorization identifies mutational signatures associated with APOBEC cytidine deaminases.

a, heatmap shows cosine similarity between six signatures identified in RRP, mucosa and HPV-associated HNSCC, using non-negative matrix factorization (columns) and COSMIC SBS prototype signatures (rows).

b, heatmap shows the relative contributions of the six mutational signatures described in **a** (columns) to explain the observed nucleotide alteration patterns within individual RRP (*n*=21), mucosa (*n*=21) and HPV-associated HNSCC (*n*=74) samples (rows). The contributions correspond to the weights that individual signatures have in the overall trinucleotide substitution matrix.

COSMIC SBS, catalog of somatic mutations in cancer – single base substitutions

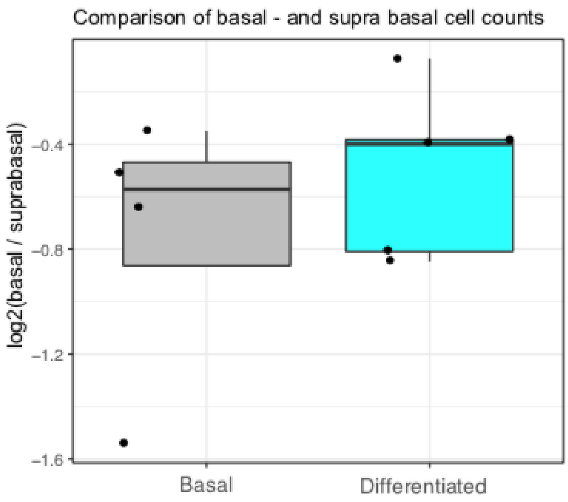

Supplementary Figure 4 - Similar proportions of basal and suprabasal cell counts in the basal and differentiated subtypes

Boxplot shows the log2-transformed ratio comparing basal to suprabasal cell counts quantified using whole-section digital pathology annotation of RRP slides from each sample stained with DAPI and pan-cytokeratin.

DAPI, 4′,6-diamidino-2-phenylindole

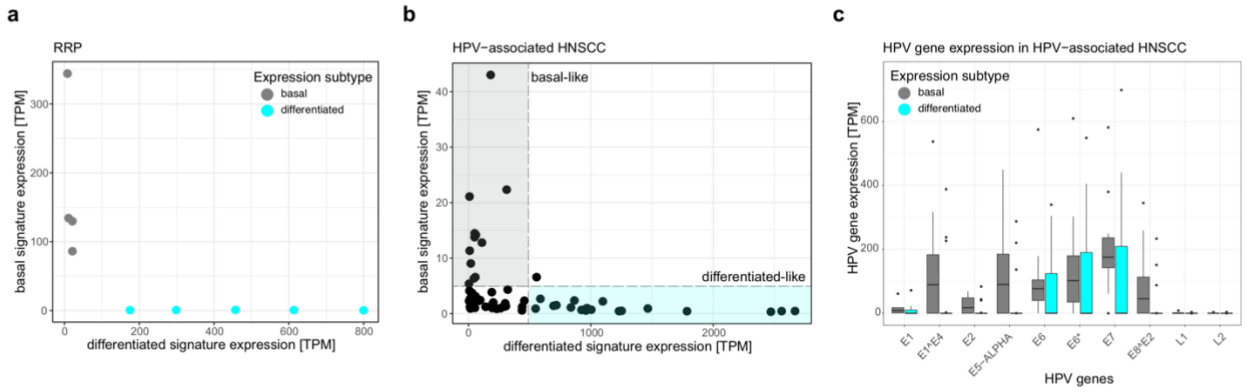

Supplementary Figure 5 - Expression of basal- and differentiated subtype signature in HPV-associated HNSCC

a, scatter plot shows the average expression the 20 most significantly upregulated genes within basal and differentiated subtype signatures in RRP samples. Color-coding is based on the PCA-based classification related **Figure 5a**.

b, scatter plot shows the average expression of basal and differentiated subtype signatures described in **a** in HPV-associated HNSCC samples. Colored rectangles comprise samples that were classified as basal (grey) or differentiated (cyan), respectively.

c, boxplot shows the distribution of HPV gene expression in basal and differentiated HPV-associated HNSCC samples identified in **b**.

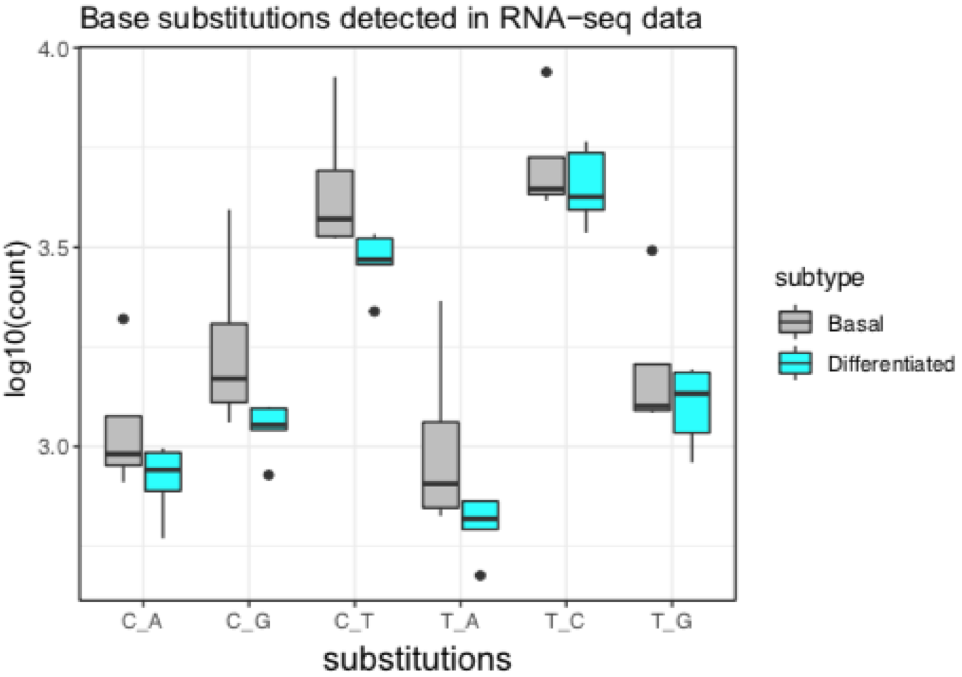

Supplementary Figure 6 - APOBEC-related substitutions are elevated in basal subtype

Boxplot shows log10-transformed substitution counts within basal and differentiated subtypes, respectively.

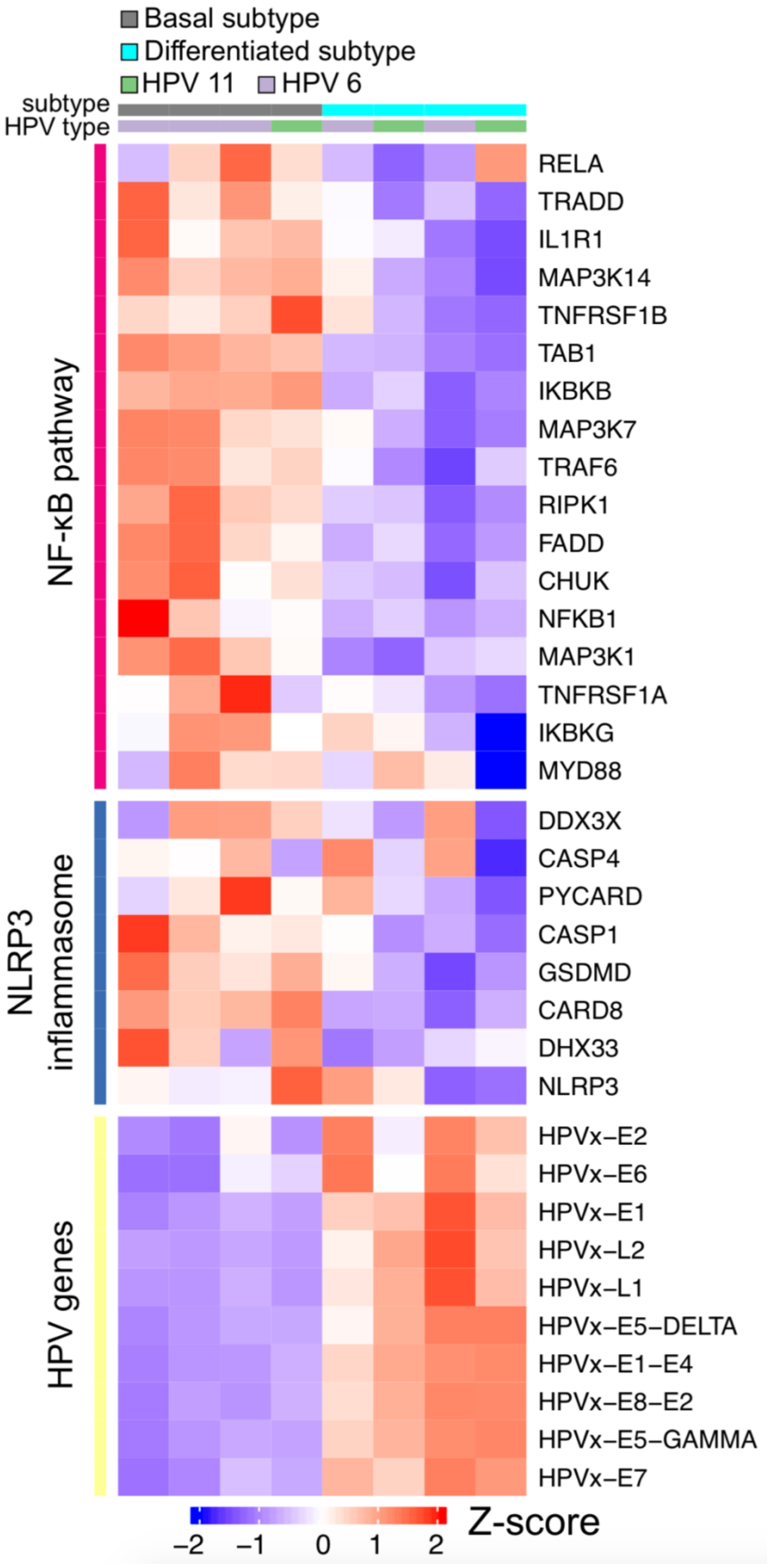

Basal subtype
Differentiated subtype
HPV 11
HPV 6
subtype
HPV type
NF-κB pathway
RELA
TRADD
IL1R1
MAP3K14
TNFRSF1B
TAB1
IKBKB
MAP3K7
TRAF6
RIPK1
FADD
CHUK
NFKB1
MAP3K1
TNFRSF1A
IKBKG
MYD88
NLRP3 inflammasome
DDX3X
CASP4
PYCARD
CASP1
GSDMD
CARD8
DHX33
NLRP3
HPV genes
HPVx–E2
HPVx–E6
HPVx–E1
HPVx–L2
HPVx–L1
HPVx–E5–DELTA
HPVx–E1–E4
HPVx–E8–E2
HPVx–E5–GAMMA
HPVx–E7
−2
−1
0
1
2
Z-score

Supplementary Figure 7 – NF-κB and inflammasome signaling are elevated in basal subtype

Heatmap shows row-standardized expression of genes related to NF-κB signaling, NLRP3 inflammasome activity and HPV gene expression (rows) within basal and differentiates subtypes. HPV type for each sample is shown above the heatmap.

Supplementary Table 1. RRP patient demographic and clinical data

Patient_id	Age at biopsy (years)	Sex	HPV type	Age at diagnosis (years)	Number of interventions in 12 mo. prior to biopsy	Number of lifetime RRP interventions	Pulmonary lesions
pt_1	32	F	11	19	3	45	No
pt_2	36	M	6	28	4	35	No
pt_3	50	F	11	5	3	275	Yes
pt_4	63	M	11	19	5	65	No
pt_5	27	F	6	6	5	65	No
pt_6	53	F	11	38	2	22	Yes
pt_7	55	M	11	53	6	11	No
pt_8	55	F	6	51	4	16	No
pt_9	56	M	11	54	8	22	No
pt_10	21	M	11	1	5	108	Yes
pt_11*	65, 67	M	11	19	6, 3	45, 55	Yes
pt_12	26	M	11	2	6	120	No
pt_13	52	F	6	23	4	74	No
pt_14	42	M	6	37	3	27	No
pt_15	37	F	6	28	8	92	No
pt_16	39	M	6	37	3	8	No
pt_17	25	M	11	1	4	112	Yes
pt_18	20	M	11	1	6	208	Yes
pt_19	18	F	6	5	6	94	No
pt_20	33	F	6	1	5	262	Yes

*Patient 11 had papilloma sampled on two different occasions

Supplementary Table 2. WES sample information.						
patient_id	sample_id	tissue_type	LCM 1=yes; 2=no	total_read_pairs	percentage_aligned	mean_coverage
pt_1	PPRE11029	Papilloma	1	77832542	99.93	146.79
pt_1	PBMC11024	PBMC	0	58456117	99.84	99.89
pt_2	NPRE11179	Normal	0	72469376	99.92	133.44
pt_2	PPRE11180	Papilloma	1	87170445	99.93	160.47
pt_2	PBMC11162	PBMC	0	51788944	99.86	89.7
pt_3	PPRE11237	Papilloma	1	70298622	99.93	131.7
pt_3	PBMC11249	PBMC	0	51901698	99.82	85.76
pt_4	NPRE11486	Normal	0	77399471	99.93	140.06
pt_4	PPRE11487	Papilloma	1	65440474	99.94	124.76
pt_4	PBMC11466	PBMC	0	54678452	99.87	99.95
pt_5	NPOS12001	Normal	0	65360789	99.88	115.84
pt_5	PPRE11558	Papilloma	1	69154460	99.92	126.56
pt_5	PBMC11556	PBMC	0	49217012	99.83	81.97
pt_6	PPRE11615	Papilloma	1	70315926	99.92	130.1
pt_6	PBMC11606	PBMC	0	52321033	99.79	76.69
pt_7	NPOS12186	Normal	0	61460298	99.84	91.47
pt_7	PPRE11691	Papilloma	1	65512991	99.89	128.83
pt_7	PBMC11675	PBMC	0	43810545	99.89	83.11
pt_8	PPRE11783	Papilloma	1	70870608	99.93	132.64
pt_8	PBMC11763	PBMC	0	52314738	99.84	95.69
pt_9	NPRE12009	Normal	0	69973891	99.95	127.14
pt_9	PPRE12010	Papilloma	1	72935969	99.95	129.49
pt_9	PBMC11991	PBMC	0	55062487	99.83	96.46
pt_10	NPRE12280	Normal	0	40216073	99.89	69.32
pt_10	PPRE12281	Papilloma	1	45954100	99.9	83.11
pt_10	PBMC12274	PBMC	0	51052091	99.83	97.46
pt_11	PPRE12611	Papilloma	1	73559062	99.91	136.25
pt_11	PBMC13268	PBMC	0	52981870	99.82	99.3
pt_12	NPRE13272	Normal	0	86830476	99.93	163.14
pt_12	PPRE13273	Papilloma	1	68688863	99.91	125.24
pt_12	PBMC12615	PBMC	0	51467016	99.85	97.82
pt_11	PPRE14551	Papilloma	0	26504712	99.73	35.97
pt_11	PBMC14619	PBMC	0	34432747	99.71	39.47

pt_13	PPRE15465	Papilloma	0	32336944	99.72	35.38
pt_13	PBMC15460	PBMC	0	32400456	99.78	43.08
pt_14	PPRE15469	Papilloma	0	28679549	99.76	39.19
pt_14	PBMC15462	PBMC	0	28869782	99.75	39.59
pt_15	PPRE14748	Papilloma	0	29832158	99.74	38.71
pt_15	PBMC14894	PBMC	0	30286197	99.74	38.31
pt_16	PPRE14670	Papilloma	0	26345910	99.74	35.13
pt_16	PBMC14684	PBMC	0	36819067	99.72	38.86
pt_17	PPRE14812	Papilloma	0	38466055	99.68	38.89
pt_17	PBMC14810	PBMC	0	28334478	99.78	36.31
pt_18	PPRE15546	Papilloma	0	43757189	99.76	36.93
pt_18	PBMC15532	PBMC	0	45747341	99.75	36.59
pt_19	PPRE15609	Papilloma	0	51518499	99.76	39.89
pt_19	PBMC15614	PBMC	0	60219753	99.76	39.61
pt_20	PPRE15777	Papilloma	0	51441908	99.74	43.23
pt_20	PBMC15775	PBMC	0	64944983	99.74	32.15

Supplementary Table 3 - RNA-seq sample information.					
patient_id	sample_id	tissue_type	LCM 1=yes; 2=no	total_read_pairs	alignable_percent
pt_1	PPRE11029	Papilloma	1	113310248	72.82
pt_2	PPRE11180	Papilloma	1	113026279	58.96
pt_4	NPRE11486	Normal	0	102979801	61.82
pt_4	PPRE11487	Papilloma	1	102347785	64.05
pt_5	NPOS12001	Normal	0	105517053	52.25
pt_5	PPRE11558	Papilloma	1	112918321	62.77
pt_9	NPRE12009	Normal	0	120662298	64.04
pt_11	PPRE12611	Papilloma	1	94161307	67.23
pt_12	NPOS13470	Normal	0	103473643	60.61
pt_12	PPRE13273	Papilloma	1	92859491	62.11
pt_11	PPRE14551	Papilloma	0	47310215	77.37
pt_13	PPRE15465	Papilloma	0	45693370	74.6
pt_14	PPRE15469	Papilloma	0	45417980	74.09
pt_15	PPRE14748	Papilloma	0	39254218	75.19
pt_16	PPRE14670	Papilloma	0	44990788	75.85
pt_17	PPRE14812	Papilloma	0	43317684	74.44
pt_18	PPRE15546	Papilloma	0	33374245	76.04
pt_19	PPRE15609	Papilloma	0	51612410	77.07
pt_20	PPRE15777	Papilloma	0	52005659	78.47
