# Supplementary material for: Comprehensive multiomic characterization of human papillomavirus-driven recurrent respiratory papillomatosis reveals distinct molecular subtypes
Source: Commun Biol. 2021 Dec 20;4:1416. doi: 10.1038/s42003-021-02942-0 (PMC8688513; doi:10.1038/s42003-021-02942-0)
Supplement: Supplementary file 3 — Description of Additional Supplementary Files [file 42003_2021_2942_MOESM3_ESM.pdf]

Description of Additional Supplementary Files

File name: Supplementary Data 1.
Description: Identified SNVs and INDELs.

File name: Supplementary Data 2.
Description: TPM values for all genes.

File name: Supplementary Data 3.
Description: Differentially expressed genes.

File name: Supplementary Data 4.
Description: Immunohistochemistry quantification.
