# Supplementary material for: Comprehensive multiomic characterization of human papillomavirus-driven recurrent respiratory papillomatosis reveals distinct molecular subtypes
Source: Commun Biol. 2021 Dec 20;4:1416. doi: 10.1038/s42003-021-02942-0 (PMC8688513; doi:10.1038/s42003-021-02942-0)
Supplement: Supplementary file 8 — Reporting Summary [file 42003_2021_2942_MOESM8_ESM.pdf]

nature portfolio

Corresponding author(s): Clint T. Allen, MD

Last updated by author(s): 11/9/2021

Reporting Summary

Nature Portfolio wishes to improve the reproducibility of the work that we publish. This form provides structure for consistency and transparency in reporting. For further information on Nature Portfolio policies, see our Editorial Policies and the Editorial Policy Checklist.

Statistics

For all statistical analyses, confirm that the following items are present in the figure legend, table legend, main text, or Methods section.

n/a	Confirmed	
☐	☒	The exact sample size (n) for each experimental group/condition, given as a discrete number and unit of measurement
☐	☒	A statement on whether measurements were taken from distinct samples or whether the same sample was measured repeatedly
☐	☒	The statistical test(s) used AND whether they are one- or two-sided<br>*Only common tests should be described solely by name; describe more complex techniques in the Methods section.*
☒	☐	A description of all covariates tested
☐	☒	A description of any assumptions or corrections, such as tests of normality and adjustment for multiple comparisons
☐	☒	A full description of the statistical parameters including central tendency (e.g. means) or other basic estimates (e.g. regression coefficient) AND variation (e.g. standard deviation) or associated estimates of uncertainty (e.g. confidence intervals)
☒	☐	For null hypothesis testing, the test statistic (e.g. F, t, r) with confidence intervals, effect sizes, degrees of freedom and P value noted<br>*Give P values as exact values whenever suitable.*
☒	☐	For Bayesian analysis, information on the choice of priors and Markov chain Monte Carlo settings
☒	☐	For hierarchical and complex designs, identification of the appropriate level for tests and full reporting of outcomes
☒	☐	Estimates of effect sizes (e.g. Cohen's d, Pearson's r), indicating how they were calculated

Our web collection on statistics for biologists contains articles on many of the points above.

Software and code

Policy information about availability of computer code

Data collection	This work describes analysis of whole exome and RNA-seq datasets generated under IRB-approved clinical studies at the NIH. The methods are comprehensibly described in the "Methods" section of the manuscript.
Data analysis	This work describes analysis of whole exome and RNA-seq datasets generated under IRB-approved clinical studies at the NIH. The methods are comprehensibly described in the "Methods" section of the manuscript.

For manuscripts utilizing custom algorithms or software that are central to the research but not yet described in published literature, software must be made available to editors and reviewers. We strongly encourage code deposition in a community repository (e.g. GitHub). See the Nature Portfolio guidelines for submitting code & software for further information.

Data

Policy information about availability of data

All manuscripts must include a data availability statement. This statement should provide the following information, where applicable:

- Accession codes, unique identifiers, or web links for publicly available datasets
- A description of any restrictions on data availability
- For clinical datasets or third party data, please ensure that the statement adheres to our policy

All data is being made available in two seperate dbGaP submissions phs002373.v1.p1 and phs002374.v1.p1

Field-specific reporting

Please select the one below that is the best fit for your research. If you are not sure, read the appropriate sections before making your selection.

☒ Life sciences ☐ Behavioural & social sciences ☐ Ecological, evolutionary & environmental sciences

For a reference copy of the document with all sections, see nature.com/documents/nr-reporting-summary-flat.pdf

Life sciences study design

All studies must disclose on these points even when the disclosure is negative.

Sample size	No sample size determination was performed. This work analyzes data generated from clinical samples from two phase I clinical studies.
Data exclusions	No data were excluded
Replication	There is one sample from each patient enrolled in each clinical study. Each sample was subjected to whole exome and RNA-seq.
Randomization	Samples were not randomized
Blinding	The bioinformatic team was blinded to clinical data and all samples were de-identified prior to analysis.

Reporting for specific materials, systems and methods

We require information from authors about some types of materials, experimental systems and methods used in many studies. Here, indicate whether each material, system or method listed is relevant to your study. If you are not sure if a list item applies to your research, read the appropriate section before selecting a response.

Materials & experimental systems

n/a	Involved in the study	
☐	☒	Antibodies
☒	☐	Eukaryotic cell lines
☒	☐	Palaeontology and archaeology
☐	☒	Animals and other organisms
☐	☒	Human research participants
☐	☒	Clinical data
☒	☐	Dual use research of concern

Methods

n/a	Involved in the study	
☒	☐	ChIP-seq
☒	☐	Flow cytometry
☒	☐	MRI-based neuroimaging

Antibodies

Antibodies used	CD4 and CD8 antibodies were used for IHC
Validation	Purchased commercially from Abcam. Validation of specificity by Abcam.

Animals and other organisms

Policy information about studies involving animals; ARRIVE guidelines recommended for reporting animal research

Laboratory animals	No animals were used in this work.
Wild animals	*Provide details on animals observed in or captured in the field; report species, sex and age where possible. Describe how animals were caught and transported and what happened to captive animals after the study (if killed, explain why and describe method; if released, say where and when) OR state that the study did not involve wild animals.*
Field-collected samples	*For laboratory work with field-collected samples, describe all relevant parameters such as housing, maintenance, temperature, photoperiod and end-of-experiment protocol OR state that the study did not involve samples collected from the field.*
Ethics oversight	*Identify the organization(s) that approved or provided guidance on the study protocol, OR state that no ethical approval or guidance was required and explain why not.*

Note that full information on the approval of the study protocol must also be provided in the manuscript.

Human research participants

Policy information about studies involving human research participants

Population characteristics	Clinical samples from two non-randomized phase II clinical studies were used, NCT02859454 and NCY03707587.
Recruitment	RRP is a rare disease. Patients were recruited for each study though interaction with their referring providers. All referred patients were considered.
Ethics oversight	Both clinical studies were IRB approved.

Note that full information on the approval of the study protocol must also be provided in the manuscript.

Clinical data

Policy information about clinical studies

All manuscripts should comply with the ICMJE guidelines for publication of clinical research and a completed CONSORT checklist must be included with all submissions.

Clinical trial registration	NCT02859454 and NCT03707587
Study protocol	Full protocol available upon request.
Data collection	Data were collected at the NIH Clinical Center in Bethesda, MD from 2016 to 2020.
Outcomes	The outcomes of these phase II clinical studies were related to treatment response. Exploratory outcomes covered study of clinical specimens.
